# Side-by-side placement and removability of a novel multi-hole self-expandable metallic stent for malignant hilar biliary obstruction

**DOI:** 10.1055/a-2767-1895

**Published:** 2026-01-28

**Authors:** Masanari Sekine, Taku Mizutani, Azumi Sato, Goya Sasaki, Naruaki Takahashi, Takeshi Uehara, Hirosato Mashima

**Affiliations:** 1 26312 Department of Gastroenterology, Jichi Medical University, Saitama Medical Center, Saitama, Japan

**Fig. 1:**
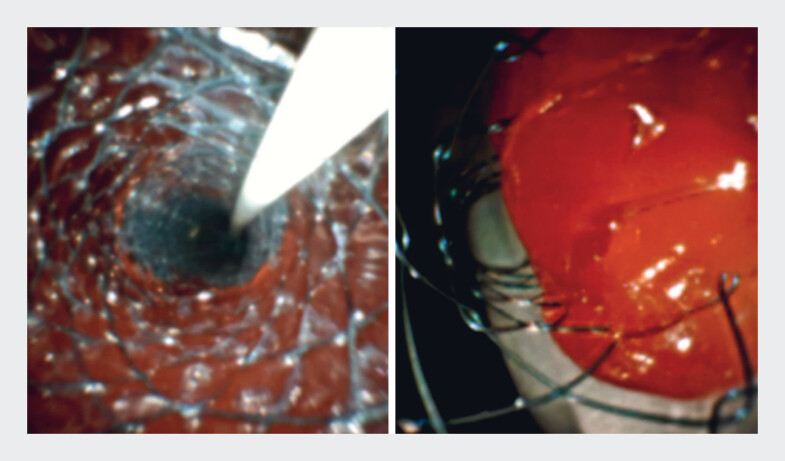
Lumen of SEMSs in a bench-top experiment. Left: A covered SEMS showing no tumor ingrowth. Right: An uncovered SEMS with complete tumor ingrowth. SEMS, self-expandable metallic stent.

**Fig. 2:**
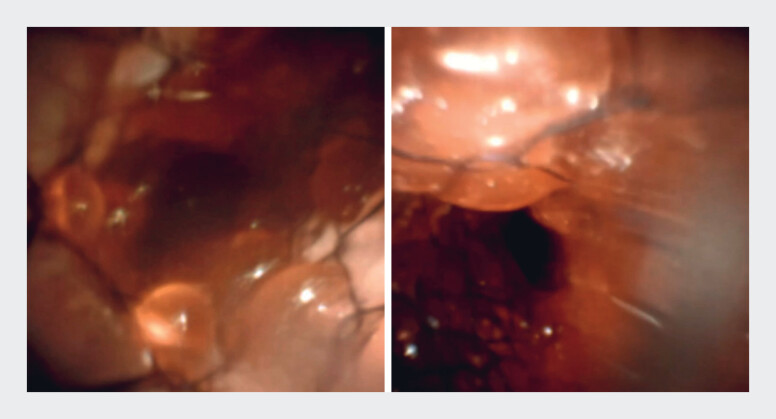
Lumen of a MH-SEMS in a bench-top experiment. Left: It showed only granular ingrowth limited to the hole regions, without bridging between them. Right: It demonstrated that, when ingrowth increases, the stent lumen becomes completely filled. MH-SEMS, multi-hole self-expandable metallic stent.

**Fig. 3:**
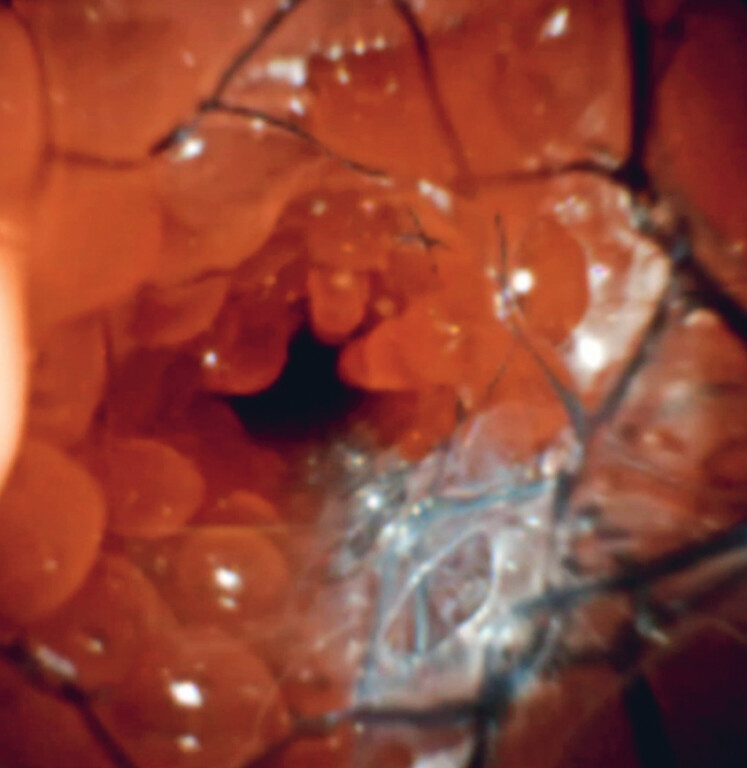
The lumen of the multi-hole self-expandable metallic stent (MH-SEMS) placed side by side in a bench-top experiment. In the lower right, the adjacent self-expandable metallic stent (SEMS) can be seen, and at the contact area, a distinct line without ingrowth is observed, representing the “non-ingrowth zone.”

For malignant hilar biliary obstruction, drainage strategies include side-by-side (SBS) or partial stent-in-stent (SIS) placement of self-expandable metallic stents (SEMSs 1 ), as well as exchangeable inside plastic stents (IS 2 ). A novel multi-hole (MH) SEMS, combining features of covered and uncovered SEMS ( Fig. 1 ), has been developed to reduce segmental cholangitis by its unique multi-hole design 3 4 . Because MH exhibits limited tumor ingrowth, stent removal is feasible; however, as tumor infiltration progresses and fills the lumen, the likelihood of difficult or impossible stent removal increases ( Fig. 2 ). A new MH variant with a 5.9 Fr delivery system and 6 mm diameter enables SBS placement 5 . The stent contact area may create a “non-ingrowth zone,” thereby limiting tumor ingrowth and facilitating removability ( Fig. 3 ).

**Fig. 4:**
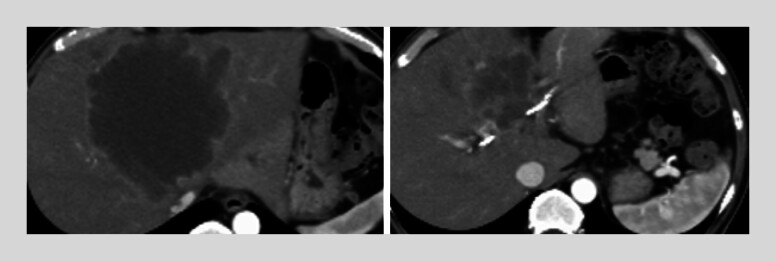
Computed tomography (CT) at presentation showing a tumor centered in hepatic segment 4 in a 59-year-old man who presented with obstructive jaundice.

**Fig. 5:**
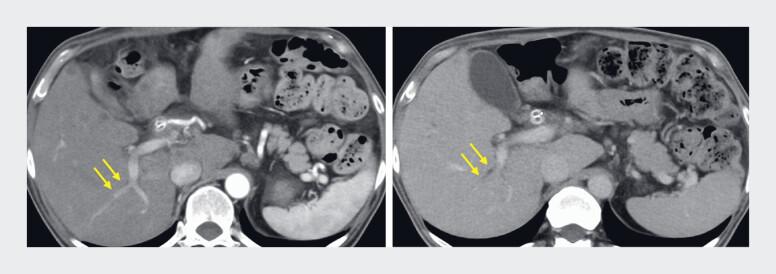
Follow-up CT obtained 49 days later during elevation of liver enzymes, demonstrating slight dilatation of bile duct limited to the posterior segment (arrow). Left: CT at the time of SBS MH-SEMS placement. Right: CT at 49 days after MH-SEMS placement. CT, computed tomography; MH-SEMS, multi-hole self-expandable metallic stent.

We present a 59-year-old man with a large hepatic segment 4 mass and obstructive jaundice ( Fig. 4 ). A plastic stent had been placed at a previous hospital. At our institution, percutaneous liver biopsy confirmed adenocarcinoma with multiple nodal metastases (intrahepatic cholangiocarcinoma cStageIVB), and systemic chemotherapy was planned. Before initiation, ERCP revealed bismuth type IIIb hilar obstruction, and two HANAROSTENT Biliary Multi-Hole Benefit stents were placed in an SBS configuration. Chemotherapy with gemcitabine, cisplatin, and durvalumab was started. Forty-nine days later, liver enzymes elevated, and posterior segmental duct dilation was noted, suggesting tumor progression ( Fig. 5 ). ERCP with stent exchange was successfully performed ( Video 1 ).

This video presents a case of side-by-side deployment and removal of a novel multi-hole SEMS, illustrating its rationale for removability and demonstrating the key technical steps required for extraction. SEMS, self-expandable metallic stent.Video 1

This video demonstrates SBS placement and safe removal of MH-SEMS, supported by bench-top experiments. SBS offers two advantages: applicability to advanced strictures (≥bismuth IIIa) and the creation of a non-ingrowth zone that improves removability. Despite a higher cost, this novel stent design may broaden therapeutic options by combining effective drainage with removability.

Endoscopy_UCTN_Code_TTT_1AR_2AZ
